# Author Correction: Plasmonic nano-aperture label-free imaging of single small extracellular vesicles for cancer detection

**DOI:** 10.1038/s43856-024-00551-6

**Published:** 2024-06-28

**Authors:** Nareg Ohannesian, Mohammad Sadman Mallick, Jianzhong He, Yawei Qiao, Nan Li, Simona F. Shaitelman, Chad Tang, Eileen H. Shinn, Wayne L. Hofstetter, Alexei Goltsov, Manal M. Hassan, Kelly K. Hunt, Steven H. Lin, Wei-Chuan Shih

**Affiliations:** 1 Department of Electrical and Computer Engineering, University of Houston, 4800 Martin Luther King Blvd., Houston, TX 77204 USA; 2 Department of Radiation Oncology, The University of Texas MD Anderson Cancer Center, 1515 Holcombe Blvd, Houston, TX 77030 USA; 3 Department of Behavioral Science, The University of Texas MD Anderson Cancer Center, 1515 Holcombe Blvd, Houston, TX 77030 USA; 4 Department of Thoracic and Cardiovascular Surgery, The University of Texas MD Anderson Cancer Center, 1515 Holcombe Blvd., Houston, TX 77030 USA; 5 Department of Epidemiology, The University of Texas MD Anderson Cancer Center, 1515 Holcombe Blvd., Houston, TX 77030 USA; 6 Department of Breast Surgical Oncology, The University of Texas MD Anderson Cancer Center, 1515 Holcombe Blvd., Houston, TX 77030 USA

**Keywords:** Cancer screening, Transmission light microscopy

Correction to: *Communications Medicine* 10.1038/s43856-024-00514-x, published online 25 May 2024

In the original version of this article, there was an error in the Results and discussion section, which read ‘Using a cutoff of 70 (3.5 ASC/µl), the post-wash sEV counts for all CPP samples (*N* = 205) were above this threshold except for one sample, and for HDP samples (*N* = 106), all but three were above this cutoff, giving a cancer detection sensitivity of 99.5% and specificity of 97.3%.’ The correct version of this sentence is ‘Using a cutoff of 70 (3.5 ASC/µl), the post-wash sEV counts for all CPP samples (*N* = 205) were above this threshold except for one sample, and for HDP samples (*N* = 106), all but three were below this cutoff, giving a cancer detection sensitivity of 99.5% and specificity of 97.3%.’ This correction has now been made in the HTML and PDF versions of the article.

