# Supplementary material for: Plasmonic nano-aperture label-free imaging of single small extracellular vesicles for cancer detection
Source: Commun Med (Lond). 2024 May 25;4:100. doi: 10.1038/s43856-024-00514-x (PMC11128000; doi:10.1038/s43856-024-00514-x)
Supplement: Supplementary file 1 — Supplementary Information [file 43856_2024_514_MOESM1_ESM.pdf]

SUPPLEMENTARY INFORMATION

Plasmonic nano-aperture label-free imaging of single small extracellular vesicles for cancer detection

Nareg Ohannesian[1†], Mohammad Sadman Mallick[1†], Jianzhong He[2], Yawei Qiao[2], Nan Li[2], Simona F. Shaitelman[2], Chad Tang[2], Eileen H. Shinn[3], Wayne L. Hofstetter[4], Alexei Goltsov[4], Manal M. Hassan[5], Kelly K. Hunt[6], Steven H. Lin[2,*], and Wei-Chuan Shih[1,**]

1 Department of Electrical and Computer Engineering, University of Houston, 4800 Calhoun Road, Houston, TX 77204, United States of America

2 Department of Radiation Oncology, The University of Texas MD Anderson Cancer Center, 1515 Holcombe Blvd., Houston, TX 77030, United States of America

3 Department of Behavioral Science, The University of Texas MD Anderson Cancer Center, 1515 Holcombe Blvd., Houston, TX 77030, United States of America

4 Department of Thoracic and Cardiovascular Surgery, The University of Texas MD Anderson Cancer Center, 1515 Holcombe Blvd., Houston, TX 77030, United States of America

5 Department of Epidemiology, The University of Texas MD Anderson Cancer Center, 1515 Holcombe Blvd., Houston, TX 77030, United States of America

6 Department of Breast Surgical Oncology, The University of Texas MD Anderson Cancer Center, 1515 Holcombe Blvd., Houston, TX 77030, United States of America

† These two authors contributed equally.

E-mail: *, shlin@mdanderson.org; **,wshih@central.uh.edu

Detection of H460 sEV on negative control IgG functionalized AGNIS surface.

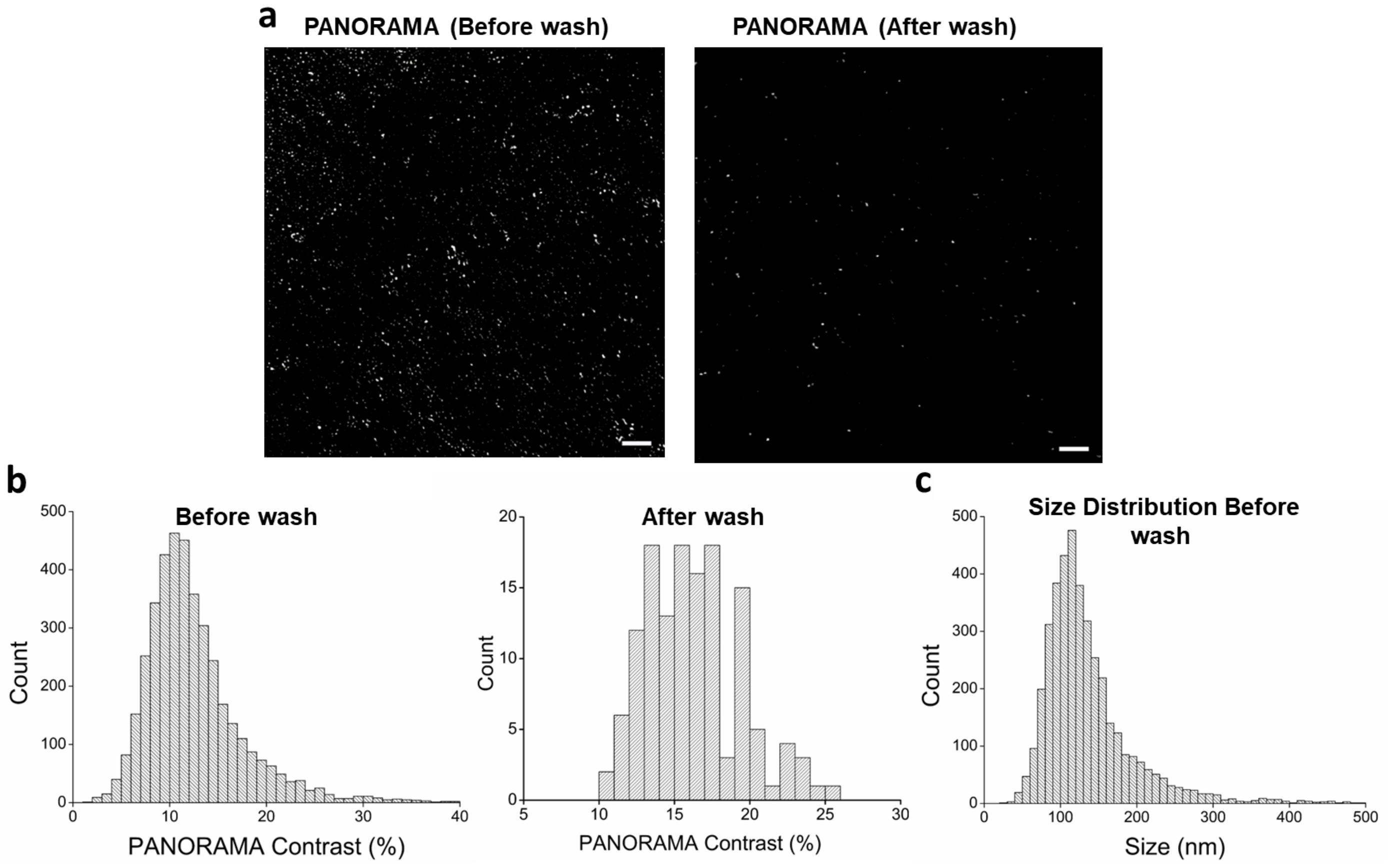

Supplementary Figure S1: Small extracellular vesicle capture via negative control using an IgG functionalized AGNIS surface. a. PANORAMA image of detected small extracellular vesicles (sEV) on negative control IgG antibody functionalized surface before and after washing with PBS. b. compiled IR histogram of detected sEV before and after wash, respectively. C. Size distribution of vesicles detected via PANORAMA on IgG surface. Scale bar: 10 µm.

Average IR value of sEV before wash was 12.54 ± 5.3 % (particle count 4044) and after wash was 16 ± 3.1 %. (particle count 135). Moreover, the average PANORAMA contrast of detected H460 sEV on IgG functionalized AGNIS surface (Fig. S1b) is highly agreeable to H460 sEV PANORAMA contrasts detected on anti-CD63 (Fig. 1e) functionalized AGNIS surfaces. Fig. S1c shows the size distribution of vesicles detected before wash with an average of 133 ± 56 nm.

Small extracellular vesicle retention from H460 (GFP positive), H460 (non-GFP), and A549 cell injected mice plasma

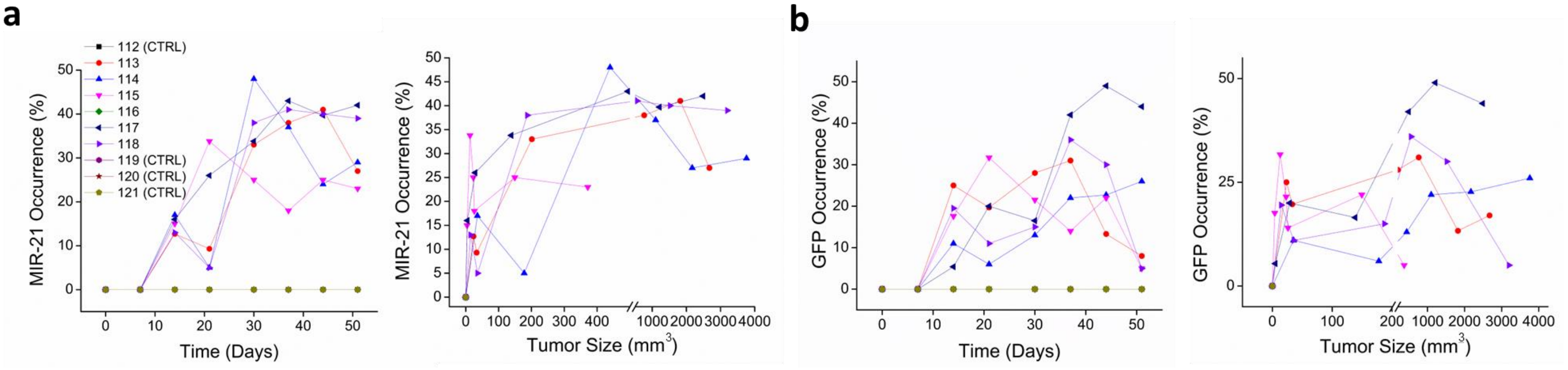

Supplementary Figure S2: Percentage of a. *miR*-21 occurrence and b. GFP occurrence among retained sEV from H460 (GFP positive) injected mice plasma.

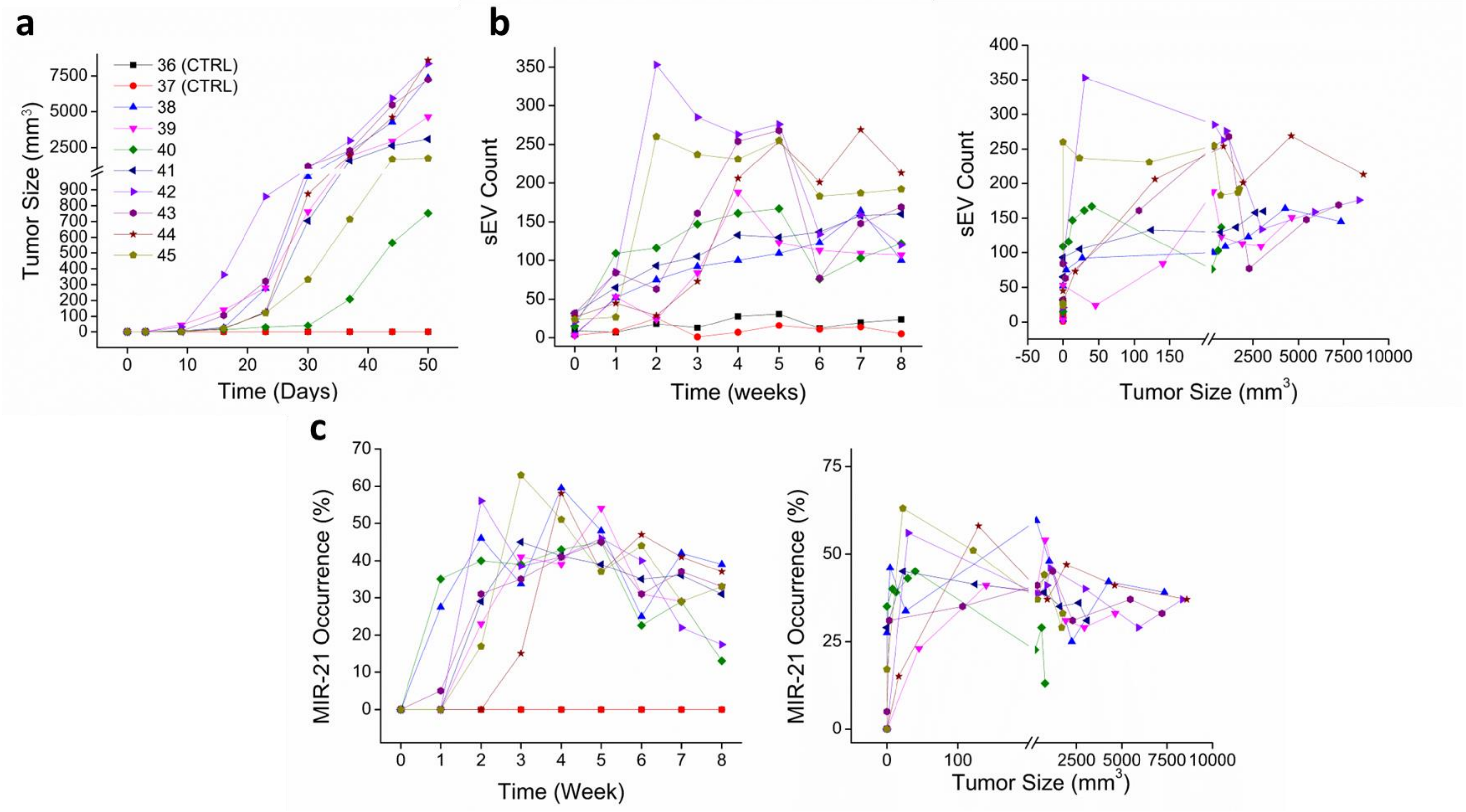

Supplementary Figure S3: a. Tumor growth vs time. b. Retained sEV and c. *miR*-21 occurrence (in %) among retained sEV from H460 (non GFP) mice plasma.

In this study, H460 (GFP negative) cancer cells were incubated in mice allowing it to grow, as seen in Fig. 3.a Plasma samples were analyzed using the same PANORAMA-Fluorescence imaging protocol from Fig. 2, AGNIS substrate functionalized with cocktail antibody mixture (anti-CD9, anti-CD63, and anti-CD81). All H460 injected mice formed a tumor size in most cases reaches sizes above 1000 mm^3 (Fig. S3a). Control plasma samples (extracted from mice #37 and #37) showed an average of 14 ± 8 retained particles and no *miR*-21 occurrence. The initial plasma drawn from remaining cancer-injected mouse plasma showed a similar range of retained sEV with no *miR*-21 occurrence. However, there was a gradual increase of both retained sEV (Fig. S3b) and *miR*-21 occurrence (Fig. S3c) as the tumor progressed within the mice. Furthermore, within three weeks of the tumor cell injection, all mouse plasma exhibited a minimum of 5-fold increase in retained particle count compared to the control values and began to show positive *miR*-21 response among retained sEV (a minimum of 17%). Furthermore, retained small extracellular vesicle count reached a plateau state as seen in H460 (GFP-positive) cell injected mice (Fig. 3). *miR*-21 occurrence was recorded among all retained sEV from cancer cell-injected mice with an average of 36% +/- 12%.

Detected particles found within healthy donor plasma detected via PANORAMA.

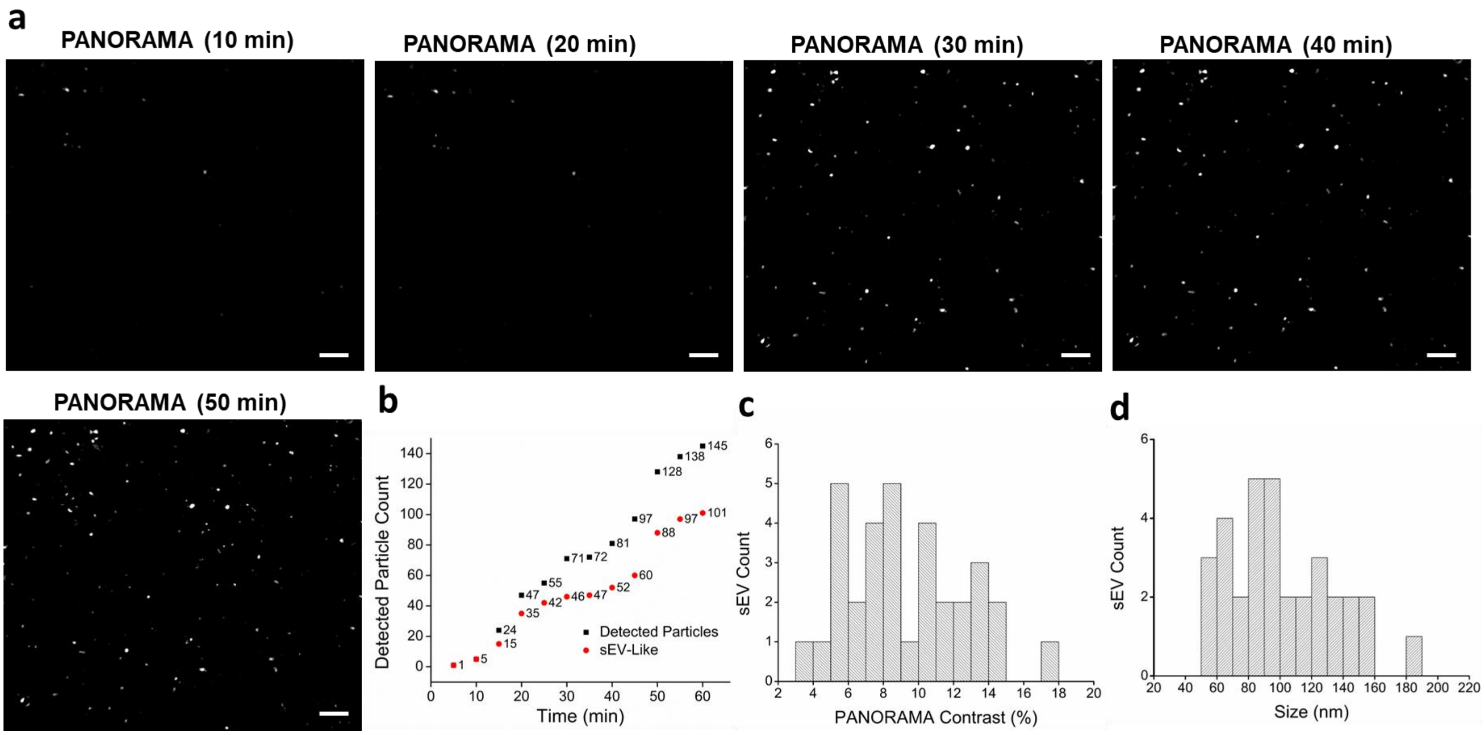

Supplementary Figure S4: a. PANORAMA image of settled plasma particles from healthy donor at times 10 min, 20 min, 30 min, 40 min, and 50 min. b. Detected particle and Small extracellular vesicle -like particle count versus time. c. PANORAMA contrast of retained sEV from healthy donor plasma. d. Retained sEV size from healthy donor plasma using PANORAMA contrast to size calibration curve. Scale bar: 10 µm.

Figure S4 is the compiled contrast histograms of retained sEV from healthy donor plasma (Fig. 4a**)**. Using the contrast to size calibration curve, the retained sEV shows an average size of 99 ± 36 nm

for the healthy patient. Retained sEV are bound to the CD9, CD81 and CD63 antibodies and exhibit a size range of sEV reported in literature (50 nm to 250 nm), thus validating the exosomal nature of bound particles.

Detected particles found within lung cancer plasma detected via PANORAMA

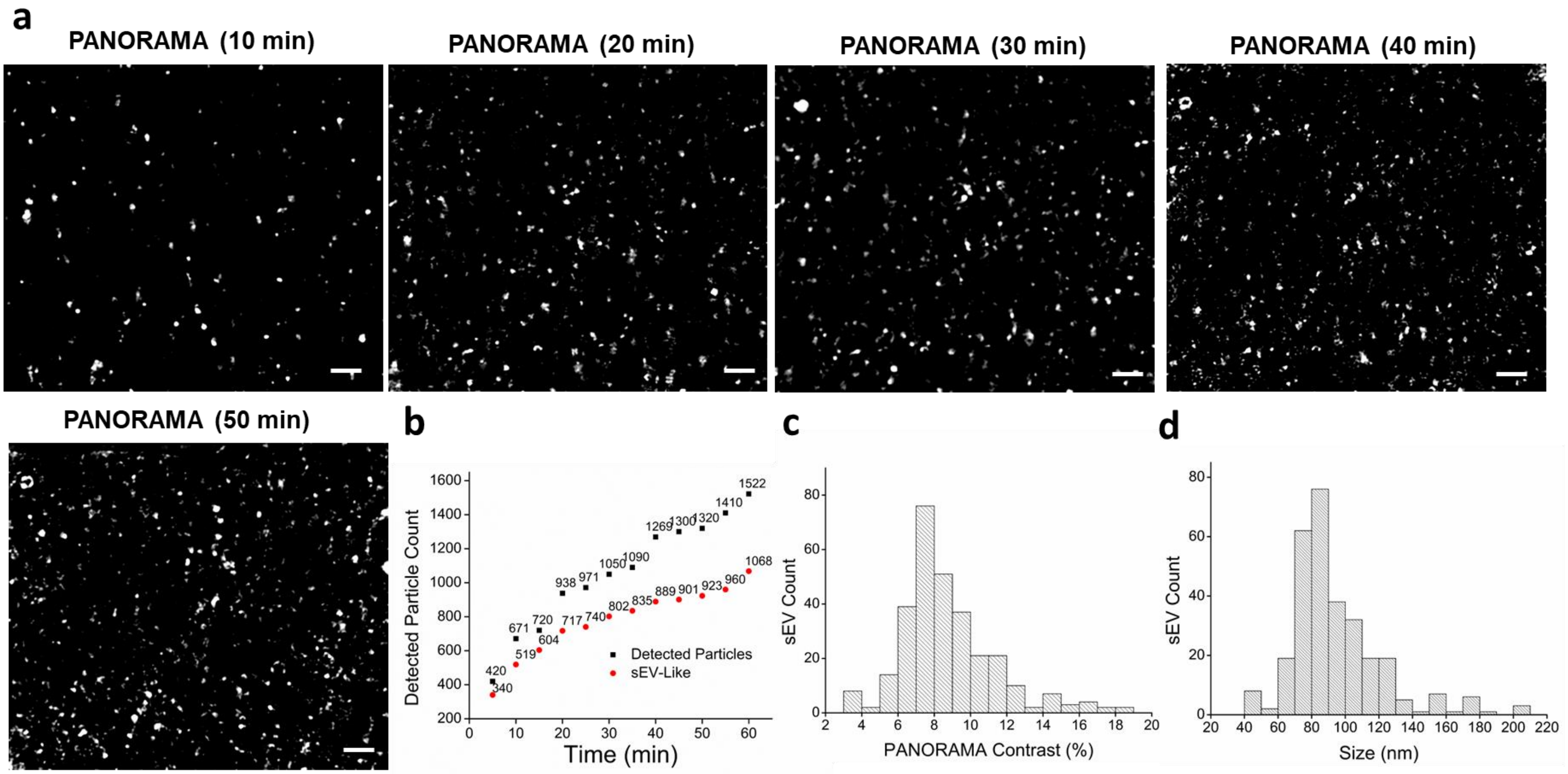

Supplementary Figure S5: a. PANORAMA image of settled plasma particles from lung cancer patient at times 10 min, 20 min, 30 min, 40 min, and 50 min. b. Detected particle and small extracellular vesicle-like particle count versus time. c. PANORAMA contrast of retained sEV from lung cancer patient plasma. d. Retained sEV size from lung cancer patient plasma using PANORAMA contrast to size calibration curve. Scale bar: 10 µm.

Figure S5 is the compiled contrast histograms of retained sEV from NSCLC patient plasma (Fig. 4b). Using the contrast to size calibration curve, the retained sEV shows a 96 ± 33 nm for NSCLC patient (Fig. S3D). Retained sEV bound to the CD9, CD81 and CD63 antibodies and exhibit a size range of sEV reported in literature (50-250 nm), thus validates the small extracellular vesicle nature of bound particles.

Retention efficiency and 99% confidence interval compiled data from plasmas in the initial cohort.

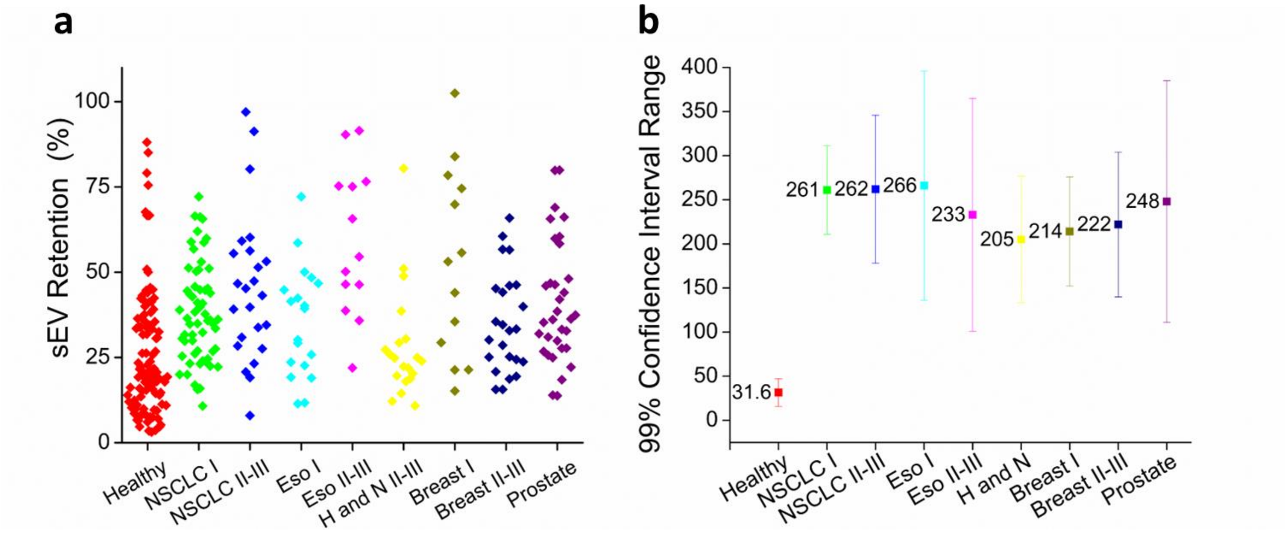

Supplementary Figure S6: a**.** Percentage of detected small extracellular vesicle retained after wash from different plasmas. b. 99% confidence interval of healthy and different cancer subsets.

ROC curve of developed protocol

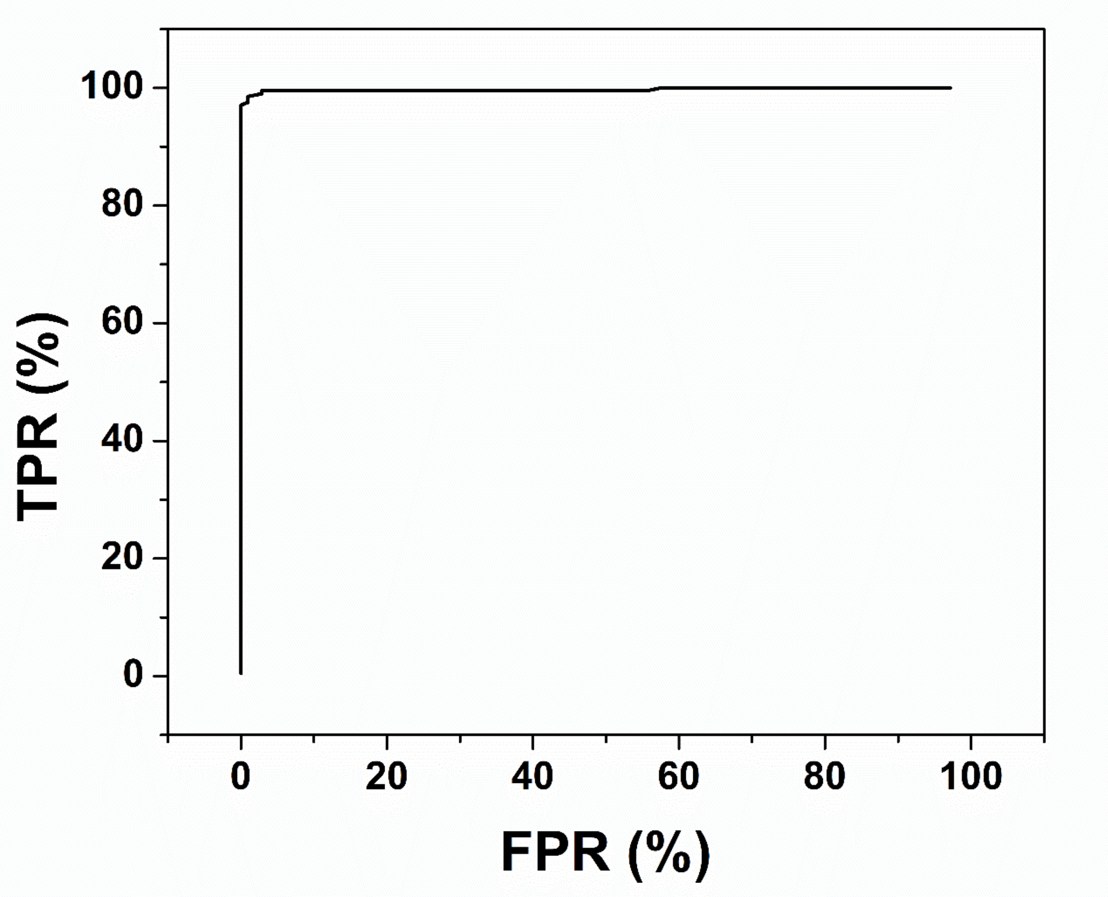

Supplementary Figure S7: Receiver-operating characteristic (ROC) curve. The area under the curve (AUC) is 96.86%.

Purified small extracellular vesicle size distribution

a

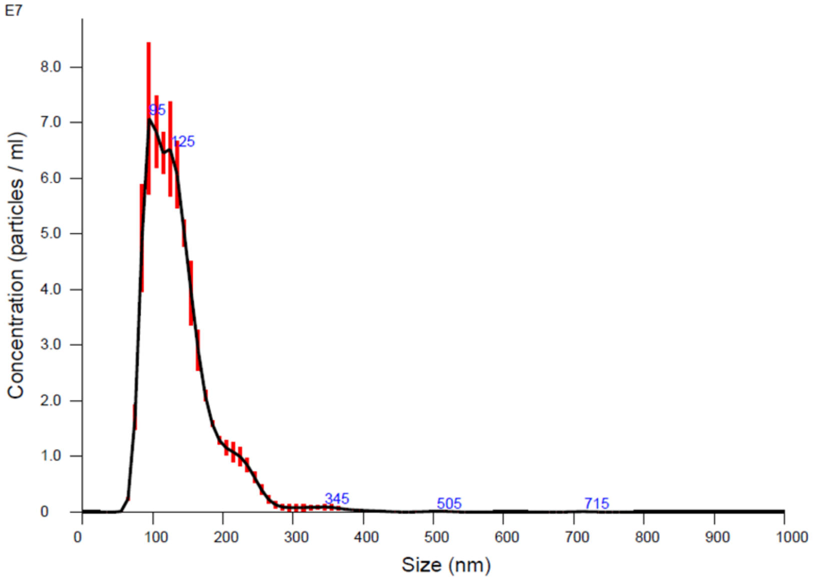

b

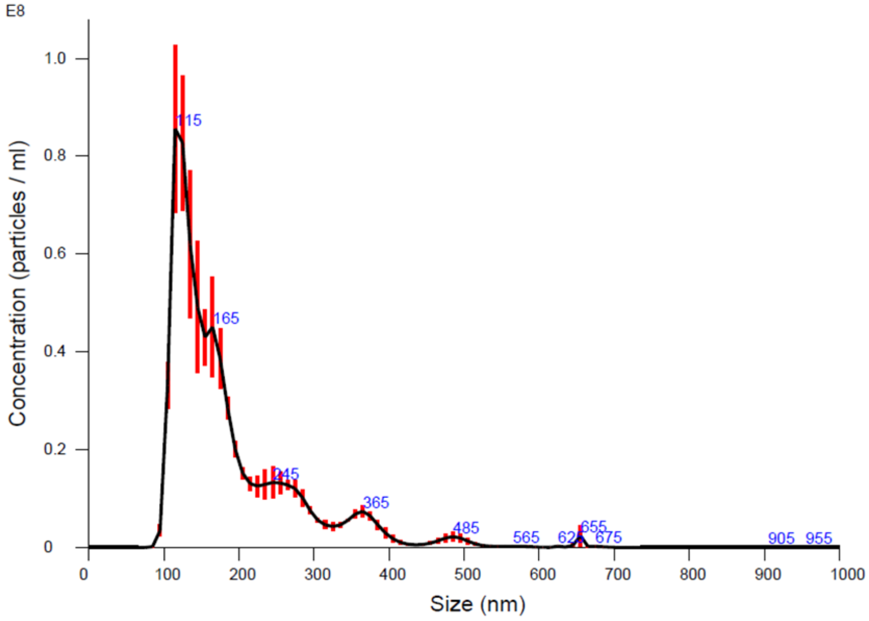

c

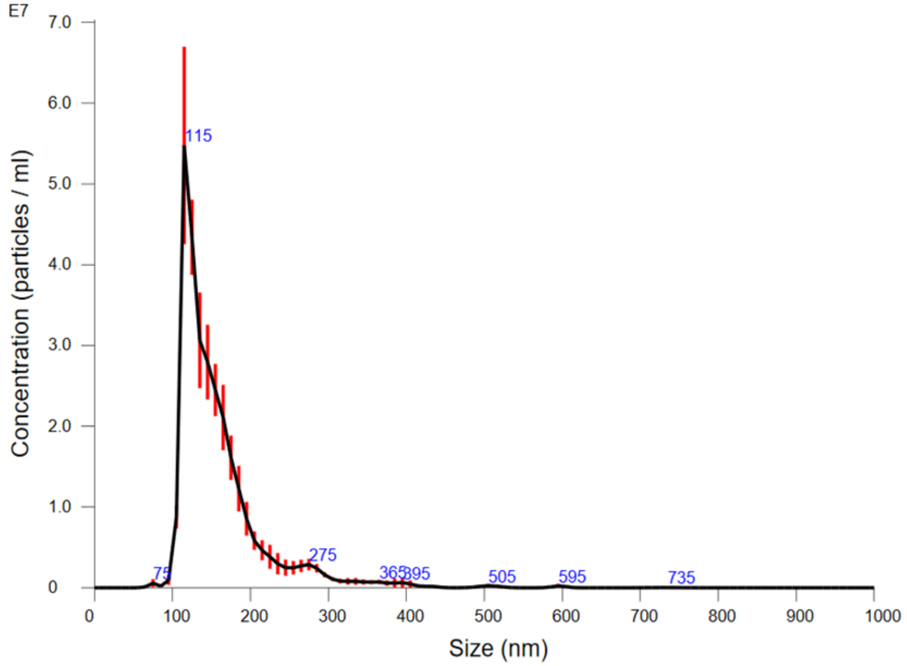

d

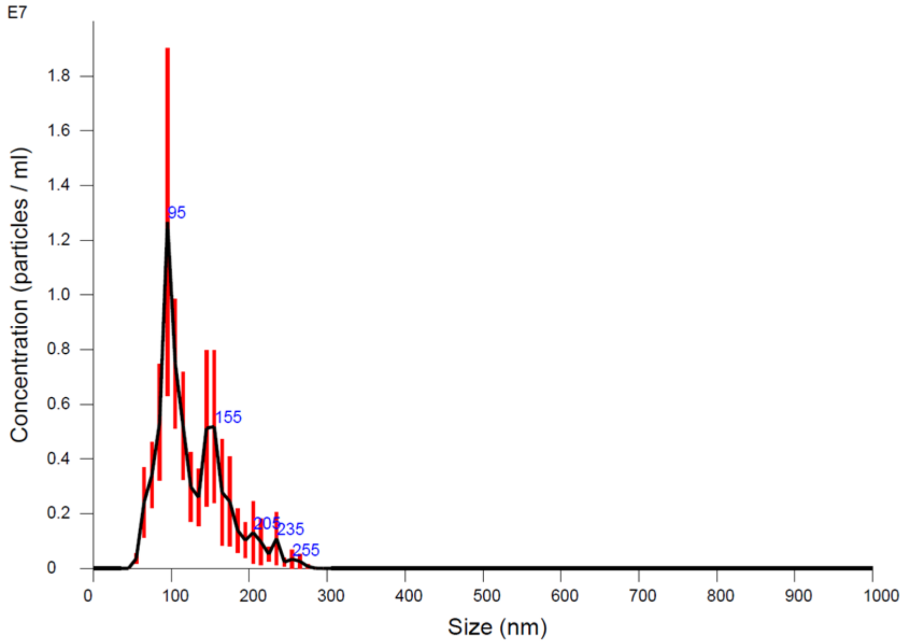

Supplementary Figure S8: Size distribution of cell line a. H460 and b. 293A c. MCF10A and d. NHBE derived sEV with respective concentration measured via Nanosight particle tracking.

Small extracellular vesicles are identified and differentiated from other extracellular vesicle types based on size. In most studies, the reported size range of sEV is between 50 to 150 nm. Thus, for validation purposes of successful small extracellular vesicle extraction, the purified solution was subjected to nanoparticle tracking analysis (NTA, Nanosight). The NTA reported a size range of 75-400 nm with most of the particles confined within the range of 50 nm and 200 nm for all small extracellular vesicle populations. The reported size range is the typical size ranges associated with sEV in literature. Furthermore, the absence of particles significantly smaller than 40 nm indicates the minimal number of ruptured sEV.
