# Supplementary material for: Plasmonic nano-aperture label-free imaging of single small extracellular vesicles for cancer detection
Source: Commun Med (Lond). 2024 May 25;4:100. doi: 10.1038/s43856-024-00514-x (PMC11128000; doi:10.1038/s43856-024-00514-x)
Supplement: Supplementary file 4 — Description of Additional Supplementary Files [file 43856_2024_514_MOESM4_ESM.docx]

**Description of Additional Supplementary Files**

**File name:** Supplementary Data 1

**Description:** Contains all the data for each figure.

**File name:** Supplementary Data 2

**Description:** Contains human plasma sample data.
