# Supplementary material for: Plasmonic nano-aperture label-free imaging of single small extracellular vesicles for cancer detection
Source: Commun Med (Lond). 2024 May 25;4:100. doi: 10.1038/s43856-024-00514-x (PMC11128000; doi:10.1038/s43856-024-00514-x)
Supplement: Supplementary file 6 — Reporting Summary [file 43856_2024_514_MOESM6_ESM.pdf]

nature portfolio

Corresponding author(s): *Double-blind peer review submissions: write DBPR and your manuscript number here instead of author names.*

Last updated by author(s): *YYYY-MM-DD*

Reporting Summary

Nature Portfolio wishes to improve the reproducibility of the work that we publish. This form provides structure for consistency and transparency in reporting. For further information on Nature Portfolio policies, see our Editorial Policies and the Editorial Policy Checklist.

Statistics

For all statistical analyses, confirm that the following items are present in the figure legend, table legend, main text, or Methods section.

n/a	Confirmed	
☐	☒	The exact sample size (*n*) for each experimental group/condition, given as a discrete number and unit of measurement
☐	☒	A statement on whether measurements were taken from distinct samples or whether the same sample was measured repeatedly
☒	☐	The statistical test(s) used AND whether they are one- or two-sided<br>*Only common tests should be described solely by name; describe more complex techniques in the Methods section.*
☐	☒	A description of all covariates tested
☐	☒	A description of any assumptions or corrections, such as tests of normality and adjustment for multiple comparisons
☐	☒	A full description of the statistical parameters including central tendency (e.g. means) or other basic estimates (e.g. regression coefficient) AND variation (e.g. standard deviation) or associated estimates of uncertainty (e.g. confidence intervals)
☐	☒	For null hypothesis testing, the test statistic (e.g. *F*, *t*, *r*) with confidence intervals, effect sizes, degrees of freedom and *P* value noted<br>*Give P values as exact values whenever suitable.*
☒	☐	For Bayesian analysis, information on the choice of priors and Markov chain Monte Carlo settings
☒	☐	For hierarchical and complex designs, identification of the appropriate level for tests and full reporting of outcomes
☐	☒	Estimates of effect sizes (e.g. Cohen's *d*, Pearson's *r*), indicating how they were calculated

Our web collection on statistics for biologists contains articles on many of the points above.

Software and code

Policy information about availability of computer code

Data collection	Images were collected using lightfield with an EMCCD manufactured by Princeton Instruments.
Data analysis	Data analysis was done using Image J for background alignment and subtraction. Statistical analysis was done using Excel built in functions.

For manuscripts utilizing custom algorithms or software that are central to the research but not yet described in published literature, software must be made available to editors and reviewers. We strongly encourage code deposition in a community repository (e.g. GitHub). See the Nature Portfolio guidelines for submitting code & software for further information.

Data

Policy information about availability of data

All manuscripts must include a data availability statement. This statement should provide the following information, where applicable:

- Accession codes, unique identifiers, or web links for publicly available datasets
- A description of any restrictions on data availability
- For clinical datasets or third party data, please ensure that the statement adheres to our policy

Findings of this research at University of Houston of Individual participant data that underlie results reported in the article after de-identification will be available for anyone who wishes to access the data for any purpose.

Human research participants

Policy information about studies involving human research participants and Sex and Gender in Research.

Reporting on sex and gender	Biologic sex information along with ethnicity and tumor stage and age information were collected for all cancer patients. Depending on the cancer type, either males (prostate cancer) or females (breast cancer) were predominant, whereas for some other cancers, such as esophageal cancer where majority (>80%) are males, there would be some predilection for specific sex based on the epidemiology of the disease. There is no selection for particular sex, ethnicity or age that entered into the study. Patients entered the study if they were presented with cancer and consented to the biomarker protocol.
Population characteristics	The cohorts were cancer patients of various types and stages of disease that were approached for research blood biomarker collection. The details of the patient cohort are provided in the supplementary table.
Recruitment	Each patient is consented on various institutional review board (IRB) approved protocols for blood collection before treatment. Blood is collected, processed and banked in the -80 C freezers until use.
Ethics oversight	MD Anderson Cancer Center Institutional Review Board

Note that full information on the approval of the study protocol must also be provided in the manuscript.

Field-specific reporting

Please select the one below that is the best fit for your research. If you are not sure, read the appropriate sections before making your selection.

☒ Life sciences ☐ Behavioural & social sciences ☐ Ecological, evolutionary & environmental sciences

For a reference copy of the document with all sections, see nature.com/documents/nr-reporting-summary-flat.pdf

Life sciences study design

All studies must disclose on these points even when the disclosure is negative.

Sample size	Initial mice cohort transplanted with H460 (GPF) cell lines included 10 mic (4 out of 10 control) with a total of 80 samples. Second mice cohort of with transplanted A549 cell line included 5 mice (2 out of 5 control) with a total of 71 samples. Healthy patient cohort included 113 plasma collected from healthy individuals. Initial cancer cohort included 227 plasma collected from individuals with a cancer diagnosis. Blind test included 39 cancer patient plasma and 29 healthy donor plasma.
Data exclusions	No data was excluded.
Replication	The protocol was applied to various plasma samples with confirmed cancer diagnosis to validate the methodology using exosome count-based cancer diagnosis.
Randomization	The plasma samples from mice were grouped based on the type of the xenograft (e.g. H460 or A549). Cancer patient plasmas were grouped based on the type of cancer. Random Healthy donor plasmas were used to compare each tested cancer patient plasma group.
Blinding	Sarcoma and Cholangicarcinoma plasma samples were blinded to the conductor of the experiments. Once all results were reported to a panel of group members, the samples were unblinded and compared to the findings.

Reporting for specific materials, systems and methods

We require information from authors about some types of materials, experimental systems and methods used in many studies. Here, indicate whether each material, system or method listed is relevant to your study. If you are not sure if a list item applies to your research, read the appropriate section before selecting a response.

Materials & experimental systems

n/a	Involved in the study	
☐	☒	Antibodies
☒	☐	Eukaryotic cell lines
☒	☐	Palaeontology and archaeology
☐	☒	Animals and other organisms
☐	☒	Clinical data
☒	☐	Dual use research of concern

Methods

n/a	Involved in the study	
☒	☐	ChIP-seq
☒	☐	Flow cytometry
☒	☐	MRI-based neuroimaging

Antibodies

Antibodies used	Anti-IgG, Anti CD9. Anti-CD63, and anti-CD81.
Validation	Relevant citation and included in the manuscript.

Animals and other research organisms

Policy information about studies involving animals; ARRIVE guidelines recommended for reporting animal research, and Sex and Gender in Research

Laboratory animals	Female nude mice
Wild animals	N/A
Reporting on sex	Female animals were used
Field-collected samples	N/A
Ethics oversight	The experiment was conducted according to oversight and approval by institutional IACUC

Note that full information on the approval of the study protocol must also be provided in the manuscript.

Clinical data

Policy information about clinical studies

All manuscripts should comply with the ICMJE guidelines for publication of clinical research and a completed CONSORT checklist must be included with all submissions.

Clinical trial registration	All protocols are non-therapy related protocols but all are IRB approved for use to consent patients. There are no Clinical Trial registration numbers. The use protocol will be attached.
Study protocol	Each protocol are biomarker collection protocols, and 2021-0368 is an IRB-approved "use" protocol that allows for the utilization of these samples from other IRB-approved protocols that are solely for collection and banking. The principle investigators for these studies are various investigators specializing in various disease sites. 2021-0368 is included in the manuscript.
Data collection	Data for each patient is collected from the electronic medical record. Patients consented for biomarker analysis and clinical data collection for research.
Outcomes	The primary endpoint is the diagnosis of the type of cancer and the stage. No other outcomes including survival analysis were collected.
